# Removal of Endobronchial Malignant Mass by Cryotherapy Improved Performance Status to Receive Chemotherapy

**DOI:** 10.1155/2014/369739

**Published:** 2014-10-15

**Authors:** Yueh-Fu Fang, Meng-Heng Hsieh, Tsai-Yu Wang, Horng-Chyuan Lin, Chih-Teng Yu, Chun-Liang Chou, Shu-Min Lin, Chih-Hsi Kuo, Fu-Tsai Chung

**Affiliations:** ^1^Department of Internal Medicine, Chang Gung Memorial Hospital at Linkou, College of Medicine, Chang Gung University, Taipei, Taiwan; ^2^Graduate Institute of Clinical Medical Sciences, College of Medicine, Chang Gung University, Taiwan; ^3^Department of Thoracic Medicine, Chang Gung Memorial Hospital, No. 5, Fusing Street, Gueishan Township, Taoyuan County 333, Taiwan

## Abstract

Although malignant endobronchial mass (MEM) has poor prognosis, cryotherapy is reportedly a palliative treatment. Clinical data on postcryotherapy MEM patients in a university-affiliated hospital between 2007 and 2011 were evaluated. Survival curve with or without postcryotherapy chemotherapy and performance status (PS) improvement of these subjects were analyzed using the Kaplan-Meier method. There were 59 patients (42 males), with median age of 64 years (range, 51–76, and median performance status of 2 (interquartile range [IQR], 2-3). Postcryotherapy complications included minor bleeding (*n* = 12) and need for multiple procedures (*n* = 10), while outcomes were relief of symptoms (*n* = 51), improved PS (*n* = 45), and ability to receive chemotherapy (*n* = 40). The survival of patients with chemotherapy postcryotherapy was longer than that of patients without such chemotherapy (median, 534 versus 106 days; log-rank test, *P* = 0.007; hazard ratio, 0.25; 95% confidence interval, 0.10–0.69). The survival of patients with PS improvement postcryotherapy was longer than that of patients without PS improvement (median, 406 versus 106 days; log-rank test, *P* = 0.02; hazard ratio, 0.28; 95% confidence interval, 0.10–0.81). Cryotherapy is a feasible treatment for MEM. With better PS after cryotherapy, further chemotherapy becomes possible for patients to improve survival when MEM caused dyspnea and poor PS.

## 1. Introduction

Previous studies show that patients with malignant endobronchial mass (MEM) and central airway obstruction of the trachea and main bronchi have poor prognosis. These patients may suffer from life-threatening dyspnea and hemoptysis and require urgent therapy. The obstructed airway caused by the neoplasm can be worsened by situations such as extreme mucous secretion and development of mucous plugs and blood and blood clots in the airway lumen [1–3].

Management of endobronchial mass with central airway obstruction remains challenging [4]. In patients with obstruction of the trachea and main stem bronchi with tumor invasion, respiratory failure is one of the most severe complications. With advances in airway recanalization techniques, interventional bronchoscopic procedures have been reported to facilitate weaning from mechanical ventilation [5, 6]. For tumors extending into the airway lumen, the primary goals of therapy are palliative relief of the malignant obstruction. Options include laser ablation, photodynamic therapy, and brachytherapy [3, 7, 8] but satisfactory results may not be immediate or lasting. Cryotherapy by flexible or rigid bronchoscopy reportedly has a long history of quick freezing (to −70°C) and removing malignant tumors [7–9].

In the past decade, endoscopic cryotherapy has gained acceptance as palliative therapy for airway complications in unresectable MEM [8]. Cryotherapy is effective for airway stenosis from both extrinsic compression and direct tumor invasion and has been useful in the treatment of MEM [3, 8, 9].

Despite reports that endoscopic cryotherapy is effective and safe for managing MEM, its objective efficacy has not been proven through large-scale studies [9, 10]. There are also no reports analyzing factors that impact on survival among postcryotherapy MEM patients. Hence, this study was conducted to clarify the role of cryotherapy in these patients, particularly in terms of complications and outcomes after cryotherapy and factors that impact on survival.

## 2. Methods

### 2.1. Design and Ethics

This retrospective study was conducted at Chang Gung Memorial Hospital, a university-affiliated hospital in Taiwan. The hospital's institutional review board approved the methodology, assurance of patient confidentiality, and project design (IRB number 100-3211B). All patients or their representatives provided informed consent prior to cryotherapy, and written consent was given by the patients for their information to be stored in the hospital database and used for research.

### 2.2. Patients

From December 2007 to December 2011, consecutive patients with malignant endobronchial mass who received cryotherapy by flexible bronchoscope were enrolled. Due toillness severity, high surgical risk, or surgical refusal, none of these patients were candidates for surgery or stent implantation under rigid bronchoscopy.

### 2.3. Collection of Clinical Data, Complications, and Outcomes after Cryotherapy

Clinical data of the study patients, including age, sex, performance status, pathologic diagnosis and locations of endobronchial masses, complications, and outcomes after cryotherapy, were obtained from chart records. Complications included minor bleeding (<200 mL and managed during the procedure), major bleeding (>200 mL or life-threatening bleeding), need for multiple procedures (residual mass necessitating repeat procedures), and pneumothorax (including mediastinal emphysema). Outcomes were relief of symptoms, improved performance status, and ability to receive further chemotherapy after cryotherapy.

### 2.4. Cryotherapy

A cryoprobe with carbon dioxide as cryogen was used. A temperature of approximately −70°C was achieved at the probe tip. All study patients received cryotherapy via flexible bronchoscopy under sedation. The principles of flexible bronchoscopy and procedure under sedation and local anesthesia in the study institution were in accordance with previous reports [11–15]. Briefly, sedation with intravenous midazolam (before 2006) or propofol under bispectral index for consciousness monitor and local anesthesia with 2% xylocaine solution was performed during bronchoscopy [15]. Oxygen saturation, blood pressure, and electrocardiography (ECG) were monitored during bronchoscopy.

**Figure 1:**
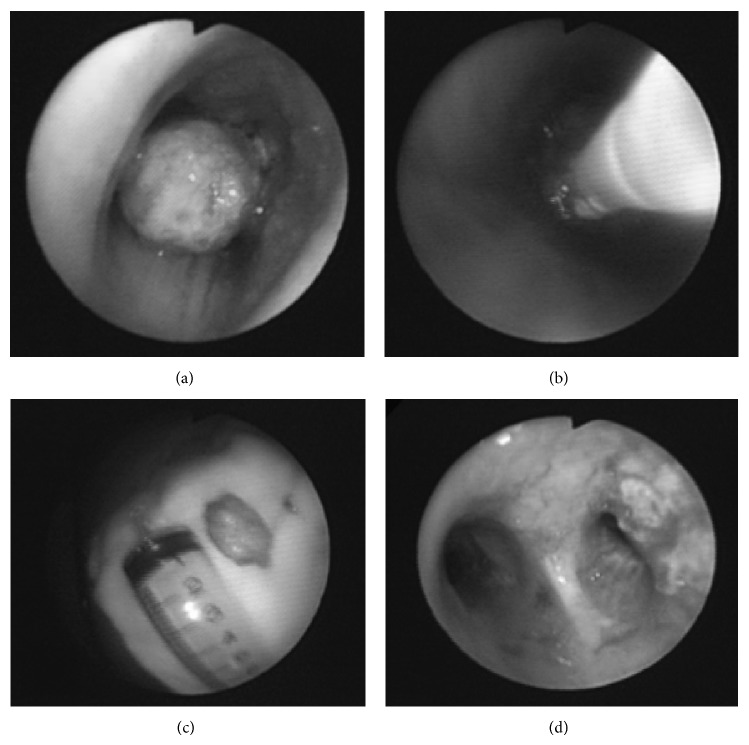
Removal of endobronchial mass by cryotherapy. (a) The bronchoscope was inserted at the proximal end of the lesion. (b) The probe was inserted via bronchoscope to contact the mass and cryotherapy was started with −70°C CO_2_ for 20–60 seconds at the lesion site. (c) After cryotherapy, the mass was detached and measured outside the patient. (d) After complete removal of the mass by cryotherapy, the bronchoscope was reintroduced to check airway patency.

The bronchoscope was advanced through a mouth guard into the tracheal and bronchial lumen, at the proximal end of the lesion (Figure 1(a)). The probe was inserted via the bronchoscope to establish contact with the mass. Cryotherapy was started with −70°C CO_2_ from 20 to 60 seconds at the lesion site (Figure 1(b)). The mass was detached outward by the bronchoscope after cryotherapy and measured once outside the patient (Figure 1(c)). After complete removal of the mass, the bronchoscope was reintroduced to check for airway patency (Figure 1(d)).

### 2.5. Assessment of Complications and Outcomes after Cryotherapy

Postcryotherapy complications included bleeding, multiple procedures necessity, and pneumothorax. Outcomes were symptom relief, improved performance status, and ability to receive further chemotherapy.

### 2.6. Multiple Procedures Necessity

For some patients with endobronchial mass, more procedures (>2) to remove the mass may be necessary. For example, large mass or multiple masses could not be removed in one procedure, residual mass could not be removed in one procedure, or the duration of the procedure was too long to remove the mass completely (usually more than 1 hour which may cause stress of patient). Because all the patients who received cryotherapy in our department were under flexible bronchoscopy, conscious sedation, and local anesthesia, we have published some articles (references) of interventional bronchoscopy including management of metallic stent complications. Similarly, conditions of these patients with MEM receiving cryotherapy may cause multiple procedures necessary individually.

### 2.7. Statistical Analysis

All data were expressed as median values and interquartile range (IQR) or numeric values (%). Statistical significance was set at *P* < 0.05. Survival days and rates were compared between the two subgroups with or without postcryotherapy chemotherapy and performance status improvement. Survival curves were traced using the Kaplan-Meier method and survival curves were compared using the log-rank test. All analyses were conducted using the SPSS software (version 13.0, SPSS, Chicago, IL) and Prism 5 for Windows (version 5.03, Graphpad Software Inc., San Diego, CA).

## 3. Results

During the study period, 59 consecutive MEM patients who received bronchoscopic cryotherapy were enrolled. Their demographic data were median age of 64 years (range, 51–76 years), male predominance (males 42, females 17), and 4 patients with the performance status score 2 before cryotherapy, compared to 55 patients with the performance status score more than 3 before cryotherapy (Table 1). The diagnosis of malignant endobronchial mass included lung squamous cell carcinoma (*n* = 23), lung adenocarcinoma (*n* = 11), metastatic colon adenocarcinoma (*n* = 7), sarcoma (*n* = 4), large cell carcinoma (*n* = 3), non-small cell lung cancer (*n* = 3), small cell lung cancer (*n* = 3), lymphoma (*n* = 2), mucoepidermoid carcinoma (*n* = 1), esophageal squamous cell carcinoma (*n* = 1), and metastatic renal cell carcinoma (*n* = 1).

Locations of the malignant endobronchial mass included the trachea (*n* = 12), left main bronchus (*n* = 12), right main bronchus (*n* = 11), right upper lobe bronchus (*n* = 11), right intermediate bronchus (*n* = 4), right lower lobe bronchus (*n* = 3), left upper lobe bronchus (*n* = 3), left lower lobe bronchus (*n* = 2), and right middle lobe bronchus (*n* = 1) (Table 2).

Postcryotherapy complications included minor bleeding (*n* = 12), multiple procedures necessity (*n* = 10), and pneumothorax (*n* = 0). There was no major bleeding. Outcomes were symptom relief (*n* = 51), improved performance status (*n* = 45), and ability to receive further chemotherapy (*n* = 40) (Table 3). Among 45 patients with improved performance status after cryotherapy, 4 patients were with performance status score 2 before cryotherapy and 41 patients were with performance status score more than 3 before cryotherapy. Among 40 patients who received chemotherapy after cryotherapy, 4 patients were with performance status score 2 before cryotherapy and 36 patients were with performance status score more than 3 before cryotherapy (Table 3). Five patients were with postcryotherapy performance status improvement but refused chemotherapy.

**Figure 2:**
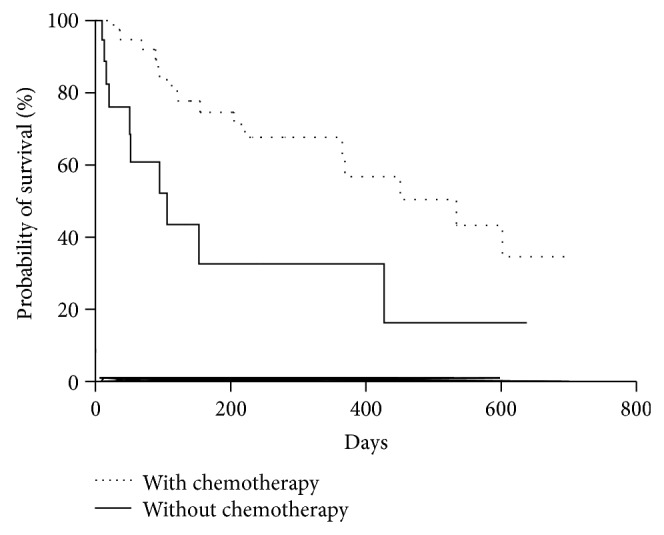
Proportion of patients who survived with and without chemotherapy traced using the Kaplan-Meier method. Median, 534 versus 106 days; log-rank test, *P* = 0.007; hazard ratio, 0.25; 95% confidence interval, 0.10–0.69.

There were significant differences between patients with and without chemotherapy after cryotherapy. By the Kaplan-Meier method, patients with chemotherapy survived longer than those without chemotherapy (median, 531 versus 106 days; log-rank test, *P* = 0.007; HR, 0.25; 95% CI, 0.10–0.69) (Figure 2).

**Figure 3:**
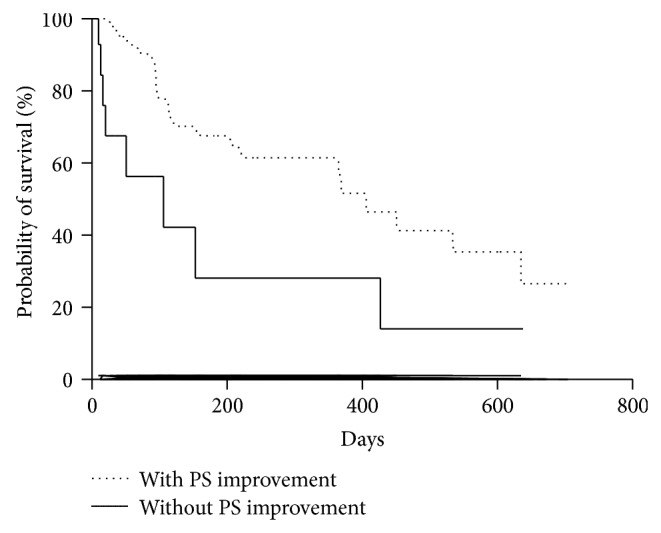
Proportion of patients who survived with and without performance status (PS) improvement after cryotherapy traced using the Kaplan-Meier method. Median, 406 versus 106 days; log-rank test, *P* = 0.02; hazard ratio, 0.28; 95% confidence interval, 0.10–0.81.

There were significant differences between patients with and without performance status (PS) improvement after cryotherapy. By the Kaplan-Meier method, patients with PS improvement survived longer than those without PS improvement (median, 406 versus 106 days; log-rank test, *P* = 0.02; HR, 0.28; 95% CI, 0.10–0.81) (Figure 3).

## 4. Discussion

Despite the poor prognosis of patients with malignant endobronchial mass, the present study demonstrates that cryotherapy is feasible and effective, especially for those who can receive chemotherapy due to improvement of performance status after cryotherapy. Patients who do not receive chemotherapy are also shown to have poor prognosis, as well as patients who may not be suited to receive cryotherapy for poor performance status.

Of the 59 MEM patients included here, 51 felt symptoms relief, 45 had improved performance status, and 40 were able to receive chemotherapy after cryotherapy. Above all, among 45 patients with improved performance status after cryotherapy, 41 (91%) patients were with performance status score more than 3 before cryotherapy. And then, among 40 patients who received chemotherapy after cryotherapy, 36 (90%) patients were with performance status score more than 3 before cryotherapy. Performance status improved after cryotherapy caused these patients to be able to receive chemotherapy! Those with chemotherapy had better survival than those without, and the possible reasons included fewer complications (i.e., bleeding, need for multiple procedures) and better outcomes (e.g., symptom relief, improved performance status). However, in the conduct of this study, emphasis was on the potential value of therapeutic cryotherapy for malignant endobronchial tumors.

Table 3 had revealed that 45 patients could have better performance after cryotherapy, and 40 patients could receive chemotherapy after cryotherapy! The most important was that 36 patients among the 40 patients who received chemotherapy indeed had improved performance status (ECOG score from 3 to less than 2) after cryotherapy! This data supported that 90 percent of these patients with malignant endobronchial mass (MEM) could have better performance after cryotherapy! In reverse, those patients did not have better performance status enough after cryotherapy; they could not receive chemotherapy! Therefore, the main cause of poor performance status of these patients to receive cryotherapy in our study (about 90%) was mass obstruction of large airway and caused breathlessness! Indeed, those patients with poor performance status and who could not improve after cryotherapy usually had other causes of poor performance status such as multiple metastases, infection, and poor nutrition. This viewpoint also suggested that the patients with these conditions may be not suitable to receive cryotherapy! With agreement of reviewer's comment, we also suggest that these patients who had MEM and dyspnea caused by MEM were the optimal candidates to receive cryotherapy!

While the usefulness of cryotherapy has been published a decade ago, advanced cancer with airway involvement is still a challenge for physicians. There are no current guidelines for cryotherapy procedures advising on the use of flexible bronchoscopy for these patients. Bleeding, multiple procedures for large-size masses, and pneumothorax may be complications during and after cryotherapy in such patients, which suggest poor prognosis.

However, through the current study, the effects of the cryotherapy by fiber-optic bronchoscopy are positive enough to relieve symptoms, improve performance status, and make further chemotherapy possible! After careful multivariate analysis, the ability to receive further chemotherapy remains an independent factor to improve survival in patients with malignant endobronchial mass after cryotherapy.

The alternative method of cryotherapy using flexible bronchoscopy is feasible in all patients of the present study. The mean time required for tumor removal is 9.7 minutes (range 5.6–31.6 min, data not shown). Although there may be other factors that impact on survival after cryotherapy, the ability to receive further chemotherapy remains a beneficial factor to survival. Moreover, there is no life-threatening complication that developed due to this procedure. The present technique also provides broader accessibility for patients unsuitable for surgery and may be a viable alternative when surgical or other equipment is not available. Above all, the improved performance status after cryotherapy may allow patients to receive further management such as chemotherapy, thereby improving their survival.

There were 19 patients who could not receive chemotherapy because their performance status did not improve. Most of them had an infiltrative mass extending from the lung parenchyma to the airways (data not listed). This means that the lung lesion was not improved after cryotherapy and as such performance status was also not improved. Thus, cryotherapy may be not suitable for patients with an infiltrative lung mass involving the airways.

Lee et al. [16] also report similar effects and safety of cryotherapy. However, the present study further mentions the possible effect of improving survival of these subjects after cryotherapy due to improved performance status and further chemotherapy. However, other methods that remove malignant endobronchial masses, including laser and photodynamic therapies and brachytherapy, are not available in the study institute. Furthermore, laser therapy is useful but may cause several complications like bleeding and perforation [8]. Photodynamic therapy is also useful but it is relatively expensive, and pre- and postoperation management are cumbersome [17]. Other endobronchial local treatment methods, including brachytherapy and electrocauterization, have their own weaknesses, such as radiation exposure and airway perforation. Lastly, other reasons, including the status of the patient's disease, experience of the specialist, and economic concerns, have to be considered in choosing these procedures [16].

The present study has limitations. First, a prospective, controlled study for these patients to receive cryotherapy was not performed. Nonetheless, there was no obvious diversity signifying that cryotherapy worsened survival. Blinded, randomized, controlled trials are hard to perform in these subjects due to clinical practices. Second, the quality of life of these patients was not recorded, only performance status in a retrospective manner. However, cryotherapy improved most symptoms and signs in this study and made further treatment possible. Third, those patients with poor performance status caused by etiology other than main airway obstruction dyspnea such as multiple metastases, infection, and poor nutrition may be not suitable to receive cryotherapy. Finally, factors that impact on survival may be more complex to analyze even though the present study reveals better outcomes through further chemotherapy after cryotherapy.

## 5. Conclusion

The current study corroborates that cryotherapy is a feasible treatment for MEM. The improved performance status after cryotherapy allows for further chemotherapy for these patients and therefore improves survival. Our study suggested that these patients who had MEM and dyspnea caused by MEM were the optimal candidates to receive cryotherapy. However, it may be not suitable for patients with an infiltrative lung mass involving the airways.

## Figures and Tables

**Table 1:** Demographic data of the study patients (*n* = 59).

Age, years	64 (51–76)
Gender, male/female	42/17
Performance status before cryotherapy	
ECOG = 2	4
ECOG > 3	55
Diagnosis of malignant endobronchial mass	
Lung SqCC	23
Lung adenocarcinoma	11
Colon adenocarcinoma metastasis	7
Sarcoma	4
Large cell carcinoma	3
NSCLC	3
SCLC	3
Lymphoma	2
Mucoepidermoid carcinoma	1
Esophageal SqCC invasion	1
RCC metastasis	1

SqCC: squamous cell carcinoma; TB: tuberculosis; NSCLC: non-small cell lung cancer; SCLC: small cell lung cancer; RCC: renal cell carcinoma.

**Table 2:** Location of endobronchial mass (*n* = 59).

Trachea	12
LM	12
RM	11
RUL	11
RIB	4
RLL	3
LUL	3
LLL	2
RML	1

LM: left main bronchus; RM: right main bronchus; RUL: right upper lobe bronchus; RIB: right intermediate bronchus; RLL: right lower lobe bronchus; LUL: left upper lobe bronchus; LLL: left lower lobe bronchus; RML: right middle lobe bronchus.

**Table 3:** Complications and outcomes after cryotherapy (*n* = 59).

Complications	
Minor bleeding	12
Major bleeding∗	0
Multiple procedures necessity	10
Pneumothorax∗	0
Outcomes	
Symptoms relief	51
Performance status improvement	45
ECOG 2 before cryotherapy	4
ECOG > 3 before cryotherapy	41
Received further chemotherapy	40
ECOG 2 before cryotherapy	4
ECOG > 3 before cryotherapy	36

^*^Those listed include complications reported from literature even without occurrence in this study.
